# A theory-informed approach to mental health care capacity building for pharmacists

**DOI:** 10.1186/1752-4458-8-46

**Published:** 2014-11-21

**Authors:** Andrea L Murphy, David M Gardner, Stan P Kutcher, Ruth Martin-Misener

**Affiliations:** College of Pharmacy and Department of Psychiatry, Dalhousie University, 5968 College Street, PO Box 15000, Halifax, NS B3H 4R2 Canada; Department of Psychiatry and College of Pharmacy, Dalhousie University, QEII HSC, AJLB 7517, 5909 Veterans’ Memorial Lane, Halifax, NS B3H 2E2 Canada; Sun Life Financial Chair in Adolescent Mental Health, Dalhousie University/IWK Health Centre, 5850 University Avenue, PO Box 9700, Halifax, NS B3K 6R8 Canada; School of Nursing, Dalhousie University, 5869 University Avenue, PO Box 15000, Halifax, NS B3H 4R2 Canada

**Keywords:** Community pharmacy, Mental illness, Behaviour Change Wheel, Consolidated Framework for Implementation Research

## Abstract

**Background:**

Pharmacists are knowledgeable, accessible health care professionals who can provide services that improve outcomes in mental health care. Various challenges and opportunities can exist in pharmacy practice to hinder or support pharmacists’ efforts. We used a theory-informed approach to development and implementation of a capacity-building program to enhance pharmacists’ roles in mental health care.

**Methods:**

Theories and frameworks including the Consolidated Framework for Implementation Research, the Theoretical Domains Framework, and the Behaviour Change Wheel were used to inform the conceptualization, development, and implementation of a capacity-building program to enhance pharmacists’ roles in mental health care.

**Results:**

The *More Than Meds* program was developed and implemented through an iterative process. The main program components included: an education and training day; use of a train-the-trainer approach from partnerships with pharmacists and people with lived experience of mental illness; development of a community of practice through email communications, a website, and a newsletter; and use of educational outreach delivered by pharmacists. Theories and frameworks used throughout the program’s development and implementation facilitated a means to conceptualize the component parts of the program as well as its overall presence as a whole from inception through evolution in implementation. Using theoretical foundations for the program enabled critical consideration and understanding of issues related to trialability and adaptability of the program.

**Conclusions:**

Theory was essential to the underlying development and implementation of a capacity-building program for enhancing services by pharmacists for people with lived experience of mental illness. Lessons learned from the development and implementation of this program are informing current research and evolution of the program.

## Introduction

People with lived experience of mental illness have inequalities and inequities in mental health care service access, delivery, and outcomes. A common strategy proposed for improving service access, delivery, and health outcomes includes utilizing the full complement and skills set of various health care professionals in the care of patients in community settings. This approach is included in mental health strategies and other strategic guidance. Through our program of research, we have been examining new ways in which pharmacists can support the bridging of gaps in the health system to improve care and outcomes, increase access to health information and services, and enhance patient system navigation of mental health and addictions care.

Pharmacists are traditionally viewed as the medication therapy experts. More recently, they have been recognized for their knowledge and skills in assuming advanced roles and scopes of practice in community and specialized clinical areas as well as in public health-related initiatives. Existing literature demonstrates that clinic and community practice-based pharmacists can improve mental health-related outcomes [1–3]. These outcomes go beyond mental health outcomes as pharmacists support physical and mental wellbeing of their patients while engaging in health promotion activities and managing chronic health problems.

In addition to improving outcomes, pharmacists in the Canadian context are generally thought to be accessible in both location and in terms of hours of operation. In Nova Scotia, Canada, much of the population lives within walking distance or a short drive from a community pharmacy [4], many of which are open on weekends, evenings, and holidays and pharmacists in these locations can be accessed in person or via telephone without the need of an appointment. This accessibility is viewed as an asset in rural and underserved areas and as well for people living with mental illnesses in communities who may require additional support.

In our program of research, one of our main goals is to design programs and interventions that capitalize on the knowledge, skills, and accessibility of pharmacists for improving mental health outcomes in communities. This requires pharmacists to engage in quality care activities that are within their scope of practice, but extend beyond medication dispensing duties, including health system navigation support and establishing robust linkages with community-based support groups. For most pharmacists, this would require a change in practice behaviour and role expectations. To support pharmacists in these activities, we recognized that an intervention would be needed to help with changing and facilitating behaviours. Numerous system, clinician, and patient factors complicate conceptualizing and preparing for evidence-informed practice change, implementing change, and sustaining changes in health care. Before implementing complex and typically expensive behaviour change interventions in practice, systematically conceptualizing the behaviours as they occur in context and the process of implementation for interventions can provide invaluable insights into variables that influence the process. Using frameworks, models, and theories to understand behaviours in context and design interventions allows for description, characterization, and contextualization of as many potential factors towards increasing success of changing behaviours and ideally, allowing for potential replication of interventions with adaptable implementation strategies in other settings. Based on this premise, we aimed to design our capacity-building intervention for improving community pharmacy-based mental health care using theory and frameworks to guide our work.

## Methods

Our process was iterative and fluid, moving between frameworks that considered implementation of interventions on a broad level to those that guided behaviour change for individuals. Many theories and frameworks are available to guide researchers in this area but we chose those that resonated with the purpose of our work. We selected a complementary set of implementation and behaviour change theories and frameworks to allow us to consider the effect of the implementation process on the design of the intervention. Many constructs among theories and frameworks for individual behaviour change and implementation have overlapping characteristics or qualities and for our purposes, they are not mutually exclusive. With our underlying philosophy that intervention implementation and intervention design for behaviour change are inextricably linked, this allowed us to consciously give priority to the process of implementation within the design of the intervention.

As can be anticipated with using these frameworks or theories, changing pharmacists’ behaviours in their care of patients with lived experience of mental illness, at a minimum, must consider not only the different pharmacists as individuals but the actions of these individuals within different inner contexts that exist in larger organizations with a host of external contextual factors. The strength and relative advantage of the intervention must be recognized, along with its potential for adaptation to the context prior to adoption must also be considered [5].

**Figure 1:**
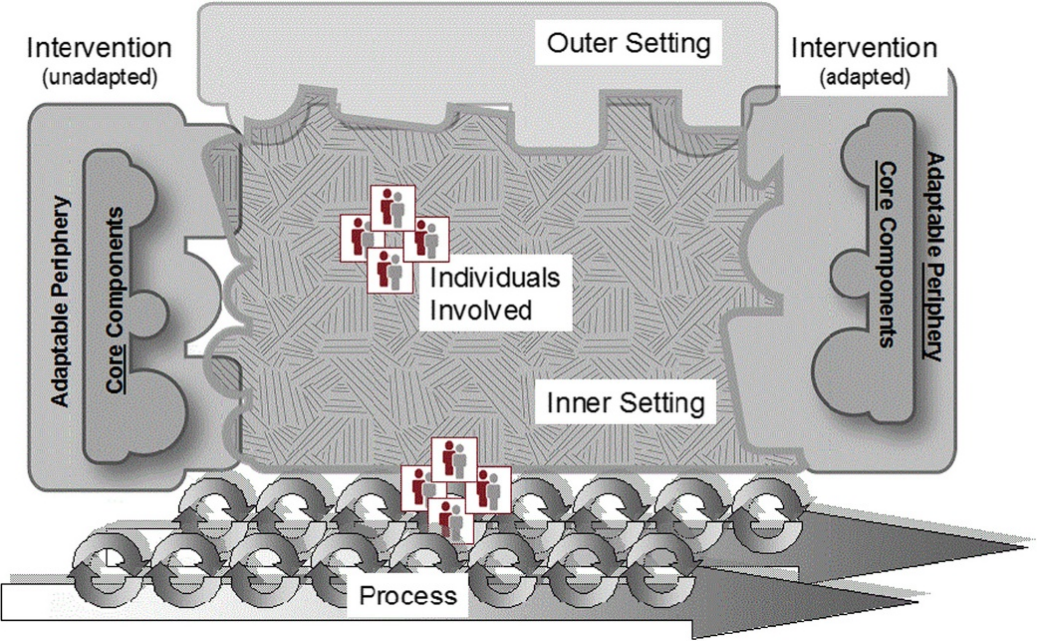
**The Consolidated Framework for Implementation Research** [5] **.**

Our first exercise included a broad examination of factors influencing pharmacists in their practice settings towards intervention development and any factors that may influence the implementation of an intervention. In our original conceptualization of an intervention, we referred to an implementation framework that we have used in our program of research previously; the Promoting Action on Research Implementation in Health Systems (PARIHS) [6–11] framework, in which successful implementation of evidence or interventions in health care will depend on the facilitation of the intervention/evidence, the context in which it is being implemented, and the strength of the evidence/intervention. However, as implementation science literature and theory continue to evolve, we found the meta-theory developed through the work of Damschroder and colleagues [5] offered advantages for us in more comprehensively conceptualizing the implementation of an intervention in pharmacy practice. The Consolidated Framework for Implementation Research (CFIR) (Figure 1), extends previous synthesis work of over 500 published articles, and was developed with the intent to create constructs for further testing and developing of theories for explaining successes and failures in healthcare implementation. The CFIR includes an additional 18 models from the published literature to create the “overarching typology” including a list of constructs to help researchers determine what works, where, and why across contexts [5]. In our work, the CFIR provided a foundation for a meta-view of understanding important variables to consider with the implementation of a complex intervention designed for changing behaviour vis-a-vis community pharmacists in mental health care. The five major domains of the CFIR include the intervention, individuals involved, the inner (including political, structural, and cultural contexts) and outer (including social, economic, and political contexts) settings, and the process by which implementation is accomplished [5]. Notably, the PARIHS framework was included in the development of the CFIR.

We then followed a step-wise approach (Table 1) to intervention design and development using the body of work by Michie and colleagues [12–17] to organize and conceptualize strategies to change behaviours. Given the current scope and stage of our research program, key behaviour changes were designed to occur at the level of community pharmacists interacting with people with lived experience of mental illness while still recognizing the many factors (e.g., inner and outer setting, intervention, etc.) that would influence the success or failures of implementing an intervention. Using our iterative and fluid process, we moved between the Behaviour Change Wheel [12] with the stepwise process to intervention development and the meta-view of implementation considerations afforded by our discussions of the CFIR [5].

**Table 1:** **Steps for designing interventions to change community pharmacists’ behaviour in mental health care** [12]

Steps	Activities
Step 1: Identify the target behaviours of pharmacists	Answer the questions: Who needs to do what differently, when, where and how?
Step 2: Understand the target behaviours of pharmacists in community pharmacy contexts	Answer the questions: Why do we do what we do? What needs to change to do things differently? Conduct a COM-B assessment (Figure 2).
Step 3: Consider the full range of interventions functions	Use the Behaviour Change Wheel (Figure 3).
Step 4: Identify specific behaviour change techniques	Use behaviour change techniques based on published evidence, if available, and tacit knowledge to support their use in changing the behaviours of pharmacists.

In the first step, the target behaviours of a typical pharmacist in a community pharmacy setting, regardless of pharmacy type (e.g., chain, independent, banner), were identified. Engaging in these discussions allowed us to detail why specific behaviours occur the way they do in practice and who needs to do what differently when, where, and how. We characterized what would be considered usual and customary pharmacist practices regarding interactions with people with lived experience of mental illness. We were informed by standards of professional practice of the time, international literature regarding practices, and more importantly, previous and ongoing contextually relevant research in our province [18–20] and our tacit knowledge regarding expectations of pharmacists and patients alike in typical encounters. At the time of intervention development, interactions between pharmacists and patients would most often occur at the time of dispensation of psychotropics, and potentially other medications for physical health problems. These discussions between pharmacists and patients would commonly include information regarding risks (e.g., undesirable effects) and benefits (e.g., time to symptom improvement) of medications. Our intention with developing an intervention was to enhance the pharmacist’s role in mental health care in a community pharmacy context, which would include practicing differently towards more actively engaging with patients for providing support in both medication-related and non-medication-related responsibilities. Examples of the latter would include: giving navigation support such as providing patients with information on local community-based resources either within or outside of the formal mental health system; providing educational outreach sessions (e.g., medications for mental and physical health conditions) for families and patients depending on needs; and overtly establishing partnerships with one or more people with lived experience of mental illness in communities to capitalize on their knowledge of community resources and relationship capital within communities.

**Figure 2:**
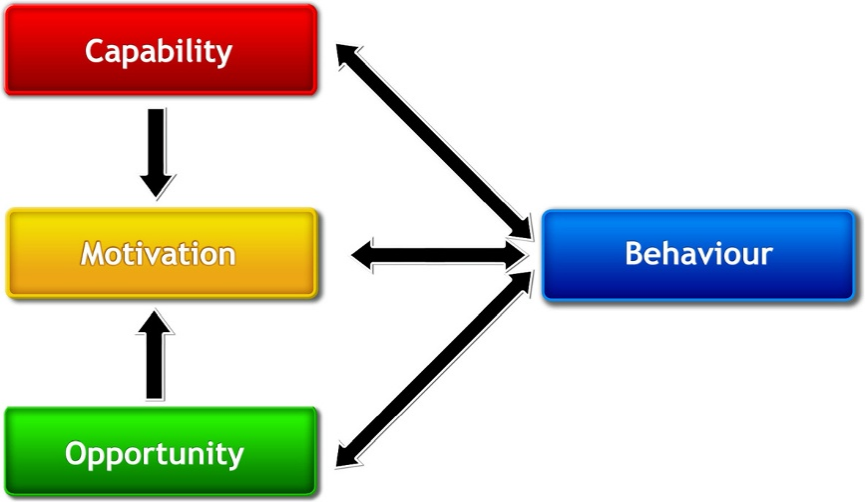
**The COM-B system: a framework for understanding behaviour** [12] **.**

As outlined in step 2 (Table 1), conducting the COM-B (C - capability, O - opportunity, M - motivation, and B – behaviour) (Figure 2) assessment, included describing the pharmacist’s capability (i.e., psychological or physical ability to enact the behaviour), motivation (i.e., reflective and automatic mechanisms that activate or inhibit behaviour such as the beliefs, intentions, plans, and wants and needs), and opportunity (i.e., physical and social environment enabling the behaviour) for behaviour change intervention design [12]. In order to complete the COM-B assessment, we continued to rely on published literature, our previous [18, 19] and ongoing research, some of which included application of the Theoretical Domains Framework, [20] and our considerable tacit knowledge of community pharmacy practice and mental health. Based on our cumulative knowledge and experience, we recognized the importance of developing an intervention that would target factors within all three components of the COM-B assessment in order to support pharmacists’ behaviour change in mental health care. As described by Michie et al., [12] and shown in the COM-B model of behaviour, each factor can influence one another such that capability and opportunity can influence motivation. Subsequently carrying out and engaging in behaviours can feedback to influence each of capability, opportunity, and motivation [12]. For pharmacists to improve services for people with lived experience of mental illness, our COM-B assessment generated many questions. Examples per component included:

Capability: Do pharmacists know how to appropriately engage people with lived experience of mental illness? Do they know how to manage and monitor patients’ pharmacotherapy and mental illness(es)?

Opportunity: Do pharmacists have access to resources to support appropriate management of conditions? Is the pharmacy equipped with a private room or area to discuss mental health concerns? Is there a stigma-free culture within the pharmacy team (e.g., pharmacists, pharmacy technicians, assistants)?

Motivations: When and how do pharmacists plan to provide services and follow-up care to people with lived experience of mental illness? Do pharmacists believe that providing improved services to people with lived experience is a good thing? Do the pharmacists have stigmatizing views? Do pharmacists want to provide these services and why? What are the incentives and disincentives to pharmacists providing improved services? What habits do pharmacists have around the existing service delivery for people with lived experience of mental illness?

**Figure 3:**
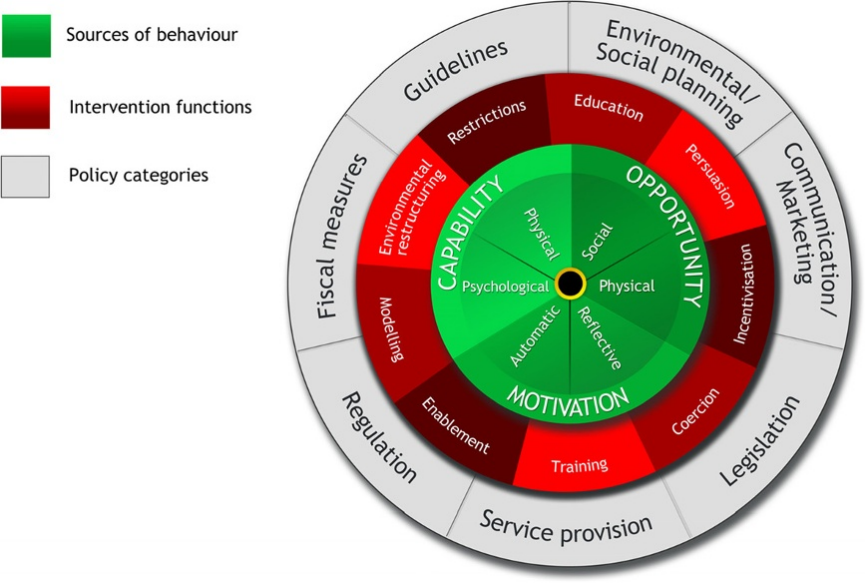
**The Behaviour Change Wheel** [12] **.**

Following the COM-B assessment in step 3 (Table 1), we considered a range of intervention functions (i.e., unique aspects of activities intended to lead to behaviour change) using the Behaviour Change Wheel for designing our intervention (Figure 3) [12]. This approach has been used and further adapted by others [21]. Michie and colleagues specifically demonstrate linkages with the COM-B model of behaviour and intervention functions [12]. We applied this in our process to determine suitable intervention functions while simultaneously conducting a “fitness for purpose” exercise within our research team. This included examining literature (published and grey) for effectiveness of suggested intervention functions and considerations of feasibility, efficiencies, and costs.

To demonstrate using one component of the COM-B assessment, capability, we discussed that pharmacists frequently report perceived deficits in knowledge and skills about how to best engage with people with lived experience of mental illness, not only with verbal communications but body language within physical spaces such as private counseling areas. In essence, the concerns reflected both physical and psychological capability of the pharmacists and based on the work of Michie et al., [12] education and training are intervention functions linked with capability. Generally, education and training sessions are standard approaches that are widely accepted by pharmacists for continuing professional education and development, including for purposes of professional licensure maintenance.

We then moved to step 4 to identify specific behaviour change techniques. We reviewed the 40 behaviour change techniques developed by Michie et al. [15, 17] and selected those deemed feasible, affordable, practical, and suitable to fit within our determined intervention functions. The expanded list of 93 behaviour change techniques was published after our intervention was designed [13] but can be considered for future interventions. Potential behaviour change techniques were also prioritized based on our knowledge and information from sources including published and grey literature [22].

Our program’s implementation subsequently moved forward with intervention implementation planning and execution by revisiting our discussions based on the CFIR. Examples of the integration with concepts explored through the CFIR and the intervention development and implementation are further discussed in the results.

## Results

Moving between applying the CFIR [5] and the Behaviour Change Wheel [12] to the development and implementation of a capacity-building, community pharmacy mental health care program (Table 2) enabled the research team to conceptualize factors and variables that could actually or potentially enable or challenge the development and implementation of the program.

**Table 2:** **Application of the Consolidated Framework for Implementation Research (CFIR) to enhancing mental health care in pharmacy practice**

Domain of CFIR	Example considerations for community pharmacy mental health care
Outer setting	Economic factors and funding model for service, belief in whether pharmacists are capable of service provision, competition and business interests within pharmacy community, general availability of mental health services.
Inner setting	Pharmacy type (e.g., chain, franchise, banner, independent, mass merchandisers & food stores), location (e.g., rural, urban), culture, computer systems, dispensary, private patient room/space, proximity and relationships with local prescribers and mental health care professionals.
Individuals involved	Community pharmacists, pharmacy technicians and assistants, front store staff, people with lived experience of mental illness and/or their support people.
Intervention	“Buy-in” from pharmacists, complexity of intervention, trialability, adaptability, relative advantage, evidence behind the intervention, quality and packaging of intervention, costs, core components such as: use of website; tools for facilitating health system navigation for patients; and community of practice communications. Adaptable periphery would include when and how to use the tools.
Intervention implementation process	Sub-processes that focus on the elements of the inner setting, individuals, adaptable intervention, and discussion regarding outer setting complexity and impact on intervention.

Many factors were considered and yet not all, especially macro considerations from the outer setting, were amenable to change within the scope of our intervention, such as political and economic influences. These factors were considered in the intervention design by addressing how pharmacists could respond in practice to these external or outer setting influences as identified in discussing the CFIR. For example, after conducting the COM-B assessment (Table 3), opportunity can influence the behaviour of pharmacists. This can manifest in the inner setting or community pharmacy practice environment as a perceived lack of time or human resources, and for some pharmacies, a lack of private space for discussions, which would require funding and renovations. These barriers to implementing new interventions in the inner setting can originate from numerous variables in the outer setting given these contexts are so closely linked as per the CFIR [5]. These factors can include, but are not limited to, economic influences such as those related to reimbursement of pharmacies for dispensing fees through private and public plans, changes in rules regarding medication manufacturer rebates, and other modifications in pharmacy business models that create change in the community pharmacy work environment. To overcome some of these implementation challenges as brought forward through examining the CFIR, the development of the intervention included discussions and examples in the education and training and throughout the program, of how to create environmental restructuring (i.e., change to the social or physical context) such as restructuring pharmacy technician and pharmacist workflow to allow for the intervention implementation, and enablement (i.e., increasing means and/or reducing barriers to increase opportunity or capability) [12] such as capitalizing on relationships with people with lived experience of mental illness in communities to facilitate determining appropriate resources that pharmacists can direct patients to accessing. Specific behaviour change techniques that were included during these exercises included: providing information on others’ approval of the behaviour; goal setting (behaviour and outcome); action planning; barrier identification/problem solving; setting graded tasks; providing instruction on when, where and how to perform the behaviour; environmental restructuring; and modeling and demonstrating the behaviour [15].

**Table 3:** **Capability, opportunity, motivation-behaviour (COM-B) assessment of community pharmacists in mental health care**

COM-B assessment	Examples for community pharmacy-based mental health care
Capability	
• Psychological and physical ability	Pharmacists can improve knowledge regarding current therapeutics and condition-specific knowledge for mental illnesses.
Motivation	
• Reflective	Pharmacists do not always recognize that people with lived experience of mental illness are not receiving the same level of service, or at times, require a higher level of service in keeping with the principle of vertical equity.
• Automatic	
	Most pharmacists intend to provide services in keeping with standards of practice.
	Pharmacist do not always plan to offer additional services to people with lived experience of mental illness.
Opportunity	
• Physical and social environment	Stigma is still a factor in community pharmacy setting within pharmacy staff that impacts care of those with mental illness.
	Barriers such as limited time, resources (including staff), and physical space limitations (e.g., lack of private area for discussion) are prevalent.

From the COM-B assessment exercise, we outlined relevant factors from published literature and our tacit knowledge regarding pharmacy practice and mental health care. Table 3 provides our examples of these factors related to our study.

The potential intervention functions identified and prioritized based on our knowledge and information from various resources are shown in Table 4.

**Table 4:** **Intervention functions for a program supporting pharmacists in providing mental health care in community pharmacies**

Intervention function	Potential model of behaviour addressed [12], [14]	Examples of current and potential strategies related to community-pharmacy based mental health care
Education	Psychological ability – capability	Education and training day on mental health including wellness and illness.
	Reflective – motivation	
Training	Psychological ability – capability	Interaction during education and training day facilitated by experts with case-based learning and simulated patients focusing on communication and management approaches for mental illness.
	Physical ability – capability	
Persuasion	Reflective – motivation	Sending pharmacists literature with statistics regarding prevalence of conditions, various services that provide benefits, and outcomes achieved with pharmacist interventions.
	Automatic – motivation	
		Use of people with lived experience of mental illness as partners in the project.
		Sending group emails with congratulatory messages to any pharmacists who did educational outreach sessions.
Incentivisation	Reflective – motivation	Using pay for performance incentives for training other pharmacists regarding the program and tools.
	Automatic – motivation	
		Community of practice inclusive of resource sharing (e.g., websites) created with group of pharmacists.
		Recognition of successes and activities through community of practice email.
Coercion	Reflective motivation	Retraction or withholding of money for lack of training of other pharmacists.
	Automatic motivation	
Restriction	Physical environment - opportunity	Rules and restriction on the kinds of services and interventions eligible for pharmacists to perform in the program.
	Social environment – opportunity	
Environmental restructuring	Automatic – motivation	Using posters in the pharmacies to advertise mental health-related services.
	Physical environment – opportunity	
		Using education and training regarding principles of the program to encourage and promote a stigma-free pharmacy culture.
	Social environment – opportunity	
		Use prompts or cues with the help of computerized systems to guide pharmacists in patient monitoring (e.g., run report of all patients taking antipsychotics and use manual or computerized prompt for pharmacists to ask patients regarding blood work or smoking status).
Modeling	Automatic – motivation	Using video clips or simulated patients demonstrating pharmacists’ assessments and monitoring of patients with mental illnesses.
	Psychological ability – capability	
Enablement	Psychological ability – capability	Providing pharmacists with access to community supports through knowledgeable people with lived experience in communities and establishing linkages in a community of practice for advice and guidance on patient-related questions.
	Physical ability – capability	Booking appointments in the pharmacies for pharmacists to spend more time with people with lived experience of mental illness.
	Physical environment – opportunity	
	Social environment – opportunity	
	Automatic – motivation	

The behaviour change techniques [13, 15, 17] primarily employed throughout our capacity building program are displayed in Table 5.

**Table 5:** **Behaviour change technique** [15] **examples to address behaviour change in community pharmacists’ provision of mental health services**

Behaviour change technique [15]	Examples of strategies used
Provide information about others’ approval	Use contact-based education with people with lived experience of mental illnesses to discuss what experiences have been positive and negative related to pharmacy practice.
Provide normative information about others’ behaviours	Discuss best practice and what pharmacists are doing for gold-standard care in mental health.
Facilitate social comparisons	Drawing attention to one or more pharmacists’ behaviours and performance in doing educational outreach.
Goal setting (behaviour)	Encourage pharmacists to make resolutions such as increasing monitoring and follow-up of patients receiving medications for mental illnesses.
Set graded tasks	Prior to delivering an educational intervention in the community, plan to perform tasks in sequence such as meet ahead of time with a community member to plan the event and organize resources prior to the event.
Action planning	Help pharmacists to develop SMART (specific, measurable, achievable, relevant, time-bound) goals around how often and when to screen for sleep problems, to use health system navigation support tools, and to work with community organizations to schedule education sessions.
Provide instruction on how to perform the behaviour	Tell and show pharmacists how to use health system navigation support tools.
Model/demonstrate the behaviour	Physically demonstrate through a case-based or simulated patient exercise the techniques for interviewing, assessing, and monitoring a patient with a mental health concern.
Provide information on where and when to perform the behaviour	Tell pharmacists about when and where to engage in related *More Than Meds* activities in their pharmacy.
Provide information on how to perform the behaviour	Discuss and use simulated patients to tell pharmacists how to perform the behaviours. Use computerized reminders on patient files to remind pharmacists about the program.
Teach to use prompts/cues	Use *More Than Meds* marketing and advertising materials in the pharmacy as a reminder to engage in the behaviours.
Use of follow-up prompts	Group emails to pharmacists reminding them of program principles, activities, and requirements.
Environmental restructuring	Alter aspects of the pharmacy to be able to accommodate *More Than Meds* materials in patient waiting area and in dispensary resource and how to better meet needs of patients with lived experience of mental illness in private counseling areas.
Time management	Discuss as a group how to manage time within workflow demands.

This process in its entirety led us to the development of *More Than Meds*. Briefly, *More Than Meds* is a capacity-building project to support pharmacists in providing enhanced mental health care for people with lived experience of mental illness. *More Than Meds* included multiple components: an education and training day that partnered community pharmacists (n = 6) with community members with lived experience of mental illness (n = 6) and eventually, 35 trained pharmacists; establishment of a community of practice via a website, regular communications, a newsletter, and the use of social media; train-the-trainer model with payments to pharmacists and community members for jointly training five other pharmacists in local or neighbouring communities; and pharmacist-led delivery of educational outreach to people with lived experience of mental illness in their communities. We collected pre and post knowledge and attitudes questionnaires; satisfaction surveys completed by pharmacists and community members who participated in the education and training components; feedback from participants attending the pharmacist-led educational outreach sessions; data regarding simulated patient consultation to community pharmacists who participated and who did not participate in the program; and qualitative interviews with an interview guide developed based on the Theoretical Domains Framework [14]. Results will be reported once the analyses are complete.

## Discussion

To our knowledge, this is the first community pharmacy-based intervention whose development and implementation was underpinned primarily by the CFIR and the work of Michie et al., including the Behaviour Change Wheel. In our experiences, the theoretical foundations of intervention development and implementation in pharmacy practice research literature is often limited or not described. There is a significant volume of research that reports on retrospective examinations of barriers and facilitators to intervention implementation in a practice change but theoretical forethought is less common, or if it exists, it is not discussed. These issues have been described by others such as Norgaard et al. [23] who encouraged debate around much of pharmacy practice research that focuses on describing the “what” and the “how many” as compared to the “why?” and “for whom?”. A recent systematic review that aimed to identify studies reporting data on pharmacists’ behaviours in clinical service provision included 21 trials of which five studies included a theoretical framework [24]. This review focused on empirical intervention studies and, as such, did not include research using other informative methods including qualitative inquiry such as those by Roberts et al. [25] or Makowsky et al. [26] or other non-quantitative study [27] and review methods [28]. These additional studies contribute knowledge in advancing the science of pharmacy practice towards inclusion of a theoretical foundation. The increased interest in a theoretical approach to explaining behaviours of pharmacists may, in part, be attributable to expanding scopes of practice including the advent of pharmaceutical care in the late 1990s. Arguably, if the pharmacy profession is to continue to move forward with expanding scopes, a theoretical basis to research will be required to advance an understanding of not only why services succeed or fail but also how to appropriately design services and implement them in community pharmacy contexts. More study will also be required to develop mechanisms for assessing context in community pharmacy practice and the work of other scholars in this area can be tested and adapted [29, 30]. It will also be important for pharmacy practice researchers to remain engaged with literature and emerging theory on intervention evolution during implementation in changing contexts. Recently, during the implementation of *More Than Meds*, sustainability of interventions was described by Chambers et al., [31] and includes the process of managing and supporting the intervention evolution within a changing context. This model of intervention implementation called the Dynamic Sustainability Framework [31] aligns with our overall approach to the mechanics of implementation with *More Than Meds* in which we attempted to maximize the fit of *More Than Meds* between practice settings and the broader ecological system through: continued learning and problem solving within our community of practice; ongoing adaptation of core components of *More Than Meds* in that we gave pharmacists freedoms and flexibilities to make needed changes in their context and considering fit with multi-level contexts; and ongoing improvements based on feedback to the research team and shared with the community of practice. An area in which we will require further development and study in our own program of research, as with much of existing pharmacy practice research, will be developing mechanisms for determining the degree of core intervention component adaptation and manipulation that can produce a desired range of effectiveness upon replication.

## Conclusion

Theory was essential to the underlying development and implementation of a capacity-building program for enhancing services by pharmacists for people with lived experience of mental illness. Lessons learned from the development and implementation of this program are informing ongoing research and the next phase of the program (http://bloomprogram.ca). As pharmacy practice advances forward with expanding scopes and increasing responsibilities in patient care activities, a theoretical foundation to developing, implementing, and evaluating services in changing contexts is necessary.

## Authors’ information

All authors are academic faculty at Dalhousie University, Halifax, Nova Scotia, Canada. We are a team of two pharmacists (AM, DG), a psychiatrist (SK), and a nurse practitioner (RMM) with an interest in advancing pharmacists’ roles in primary health care in communities with a focus on mental health care.

## Abbreviations

CFIR: Consolidated Framework for Implementation Research
PARIHS: Promoting Action on Research Implementation in Health Systems
COM-B: C - capability, O - opportunity, M - motivation, and B – behaviour.
